# Seroprevalence of Hepatitis B virus surface antigen among African blood donors: a systematic review and meta-analysis

**DOI:** 10.3389/fpubh.2024.1434816

**Published:** 2024-10-21

**Authors:** Angelina Edna Quintas, Nelson Cuboia, Lemuel Cordeiro, António Sarmento, Luís Azevedo

**Affiliations:** ^1^Department of Community Medicine, Information, and Health Decision Sciences (MEDCIDS), Faculty of Medicine, University of Porto, Porto, Portugal; ^2^Health Research Network Associated Laboratory (RISE), CINTESIS – Center for Health Technology and Services Research (CINTESIS), University of Porto, Porto, Portugal; ^3^Department of Education Office, Clínica Girassol, Luanda, Angola; ^4^CHUSJ, Infectious Diseases Service at the University Hospital Center of São João, Porto, Portugal

**Keywords:** blood donors, seroprevalence, serologic tests, Hepatitis B virus, African countries

## Abstract

**Background:**

Transfusion Transmitted Infections (TTIs) are still a growing public health problem in Africa. Studies that synthesize the available evidence on the seroprevalence of Hepatitis B Surface Antigen (HBsAg) among African blood donors are scarce. Therefore, this study aimed to synthesize qualitatively and quantitatively the seroprevalence of Hepatitis B Virus Surface Antigen (HBsAg) among blood donors in Africa.

**Methods:**

We conducted a systematic review and meta-analysis where we included all studies that reported the seroprevalence of HBsAg among blood donors in Africa. The references were searched from electronic databases: PubMed, Web of Science, Cochrane, Scopus, WHO research database-HINARI, Global Index Medicus and ClinicalTrials.gov. We further analyzed the full list of references of all included studies. The pooled seroprevalence was estimated through random effect model. The heterogeneity was assessed through Cochrane’s *Q* test and *I*^2^, respectively. Meta-regression, subgroup and sensitivity analyses were conducted.

**Results:**

We obtained 124 studies that met our inclusion criteria, comprising 3,573,211 blood donors tested for HBsAg. The pooled seroprevalence of HBsAg among blood donors in Africa was 6.93% (95% CI: 5.95–7.97%; *I*^2^ = 100%; *p* < 0.001). We found that the heterogeneity was explained by the study performed country and, African region. The higher prevalence was observed in Western 10.09% (95% CI: 8.75–11.50%), Central 7.81% (95% CI: 5.34–10.71%), and Eastern African region 4.87% (95% CI: 3.77–6.11%) and lower prevalence were observed in Southern 2.47% (95% CI: 0.54–5.75%) followed by Northern Africa region with 1.73% (95% CI: 0.45–3.79%). Additionally, based on the date of publication, we found that the highest prevalence was observed in studies published between 2001 and 2010 (9.41, 95% CI: 7.19–11.90) and the lowest prevalence was observed in studies published between 2011 and 2024 (6.26%; 95% CI: 5.19–7.42).

**Conclusion:**

The seroprevalence of HBsAg among blood donors in Africa is still very high and heterogeneous. Therefore, intensifying the screening and vaccination of the population for Hepatitis B is critical to ensure blood safety toward eliminating Hepatitis B in Africa.

**Systematic review registration:**

https://www.crd.york.ac.uk/prospero/display_record.php?RecordID=395616, PROSPERO CRD42023395616.

## Introduction

Hepatitis B Virus (HBV) remains one of the most serious public health concerns challenging the world, with an estimated 257–291 million individuals having chronic Hepatitis B (1). Africa is one of the highest-burden regions for Hepatitis B, where it is estimated that nearly 116 million people live with Hepatitis B and 81 million are chronically infected (2). An infected person can transmit HBV through direct contact with blood, unprotected sexual intercourse, use of contaminated needles and syringes, mother to child transmission during delivery, and transfusion of infected blood (3). Transfusion of infected blood is one of the main modes of HBV transmission, particularly in the sub-Saharan Africa region (4). Therefore, the World Health Organization (WHO) recommends that all countries provide access to screening and preventive measures such as vaccination and treatment for Hepatitis B (5).

Blood transfusion can be potentially lifesaving, but the risk of several Transfusions-Transmissible Infections (TTIs) such as Hepatitis B is high. For this reason, screening of blood donors for TTIs is essential for transfusion safety.

Although more sensitive tests are highly recommended for screening Hepatitis B among blood donors, most of lower- and middle-income countries still widely use rapid diagnostic tests. These methods are still indispensable to guarantee blood donation safety in many African countries (6). To maintain a safe supply of blood transfusion and products, the WHO recommends that all blood donations be screened for infections before use (7).

Several systematic reviews and meta-analyses have estimated the prevalence of hepatitis B among blood donors in some specific African countries (8–12). However, comprehensive studies on the prevalence of Hepatitis B among blood donors in Africa are scarce. Therefore, this study aimed to systematically synthesize the available evidence on the seroprevalence of Hepatitis B Virus Surface Antigen (HBsAg) among Blood Donors in Africa.

## Methods

### Study design

This study is a systematic review and meta-analysis based on The Preferred Reporting Items for Systematic Reviews and Meta-Analysis (PRISMA Statement Guideline updated in 2020) (13). The study protocol was registered in the PROSPERO with the number CRD42023395616.

### Search strategy and study selection

We included primary studies published in any language from inception through March 1st 2024, and having extractable data on seroprevalence of HBsAg among blood donors in Africa aged 16–65. We excluded case series, reviews, comments, editorials, and studies with duplicate data.

All relevant articles were searched in electronic databases, namely: PubMed/Medline, SCOPUS, Web of Science, WHO research database-HINARI, Cochrane database library, Global Index Medicus and Clinicaltrials.gov. The research query is in the Supplementary Table S1. We further analyzed systematically the full list of references of all included studies.

Two reviewers (AEQ, NC) carried out the study selection process independently, and discrepancies were resolved by the third reviewer (LA). This study was part of a more extensive research project that assessed the seroprevalence of Serologic Markers of Hepatitis B Virus (HBV), Hepatitis C Virus (HCV), Human Immunodeficiency Virus (HIV), and Syphilis in Blood Donors in Africa.

Due to the considerable volume of results, we decided to split such a study into four separate analyses based on the transmitted blood infection disease (Hepatitis B virus, Hepatitis C Virus, Syphilis, and HIV).

### Data extraction

Two reviewers, AEQ and NC independently extracted the data for each included study based on a predefined and agreed-upon data extraction form designed for this study. The differences in extracted data were discussed, and persistent discrepancies were resolved by a third reviewer (LA). For each included study, we extracted the following information: Author name, year of publication, date of participant enrolment, study design, name of the country and African region where the study was performed, the total number of participants for each study, the total number of blood donors who tested positive for HBsAg, age, sex, type of blood donors (VNRBD-Voluntary Non-Remunerated Donors, RD- Replacement or Paid Donors/FD and FD-Family Donors), and the method used for screening and Hepatitis B diagnosis. This data was stored in a Microsoft Excel 2021 spreadsheet (Microsoft Corporation, Redmond, Washington, USA).

### Study’s quality assessment

Two reviewers, AEQ and NC, independently assessed the quality of each included study using the risk of bias tool SeroTracker-RoB: a decision rule-based algorithm for reproducible risk of bias assessment of seroprevalence studies (14). The differences in the quality assessment of the included studies were discussed, and persistent disagreements were resolved by the third reviewer (LA). This tool derives from the Joanna Briggs Institute Checklist for Prevalence Studies and asks nine questions to assess the risk of bias. The questions are (a) Was the sample frame appropriate to address the target population? (b) Were study participants recruited in an appropriate way? (c) Was the sample size adequate? (d) Was the data analysis conducted with sufficient coverage of the identified sample? (e) Were valid methods used for the identification of the condition? (f) Was the condition measured in a standard, reliable way for all participants? (g) Was there appropriate adjustment for test characteristics? (h) Was there appropriate adjustment for population characteristics? (i) Was the response rate adequate, and if not, was the low response rate unlikely to introduce bias? And the last was the assessment of the overall risk of bias (lower, moderate, high and unclear) according to the scores from the responses of the previous nine items.

### Data analysis

All the data were analyzed through R software version 4.3.2 (2023-10-31) using meta package and the functions for meta-analysis of proportion (15). We used the proportion of blood donors who tested positive for HBsAg as the parameter of interest to be estimated as our effect measure and meta-analyzed. We used the DerSimonian-Laird random effects model to estimate the pooled seroprevalence of HBsAg among blood donors in Africa, and the proportions were estimated based on Freeman-Tukey double arcsine transformation (FTT) (16). The findings were presented with 95% confidence intervals.

We run a Cochrane Q test and I^2^ statistic (percentage of total variability due to true heterogeneity, that is, to between-studies variability) to assess the presence of heterogeneity and its relative magnitude, respectively (17). We performed subgroup and sensitivity analysis to investigate the moderator variables of the observed heterogeneity. Because we are analyzing and synthesizing prevalence studies from all of Africa and several different countries, we inherently assumed the presence of heterogeneity, and we mainly focused our analysis and results on subgroups and the assessment of moderators of heterogeneity.

The subgroup analysis studies were stratified by the country, African region, and year of publication. The years of publication were categorized into three categories (before 2000, 2001–2010 and 2011–2024). This cut-off was chosen based on the behavior of the distribution of the number of studies by year. To determine the moderators of heterogeneity, temporal trends and regional differences in our study, we performed meta-regression analyses using the following variables: year of study publication and African region (Western, Northern, Eastern, Central, and Southern), risk of bias, study location (unicentric and multicentric), setting (Urban and Rural), proportion of men, age, type of blood donors and country where the study was performed. In our study, we defined study location as unicentric if the study was carried out in a single center or one hospital. In contrast, a multicentric study means the study was conducted in multiple centers or hospitals. The setting variable refers to whether the study was conducted in an urban or rural area.

The publication bias was assessed through a funnel plot and by Egger’s statistics regression test. We mapped the spatial pattern of the pooled estimates of seroprevalence of HBsAg among blood donors in Africa by country. The map was created using Quantum Geographic Information System (QGIS) software (18).

## Results

A total of 4,408 were identified through database and manual searching, and 500 duplicate articles were removed. The title and abstract of the remaining 3,908 were screened, and 3,605 articles were removed as they were found to be irrelevant to our study. The remaining 303 references were assessed for eligibility through the complete text examination, and 179 were excluded because they did not meet our inclusion criteria. The remaining 124 studies were considered for qualitative and quantitative synthesis involving 3,573,211 participants.

**Figure 1:**
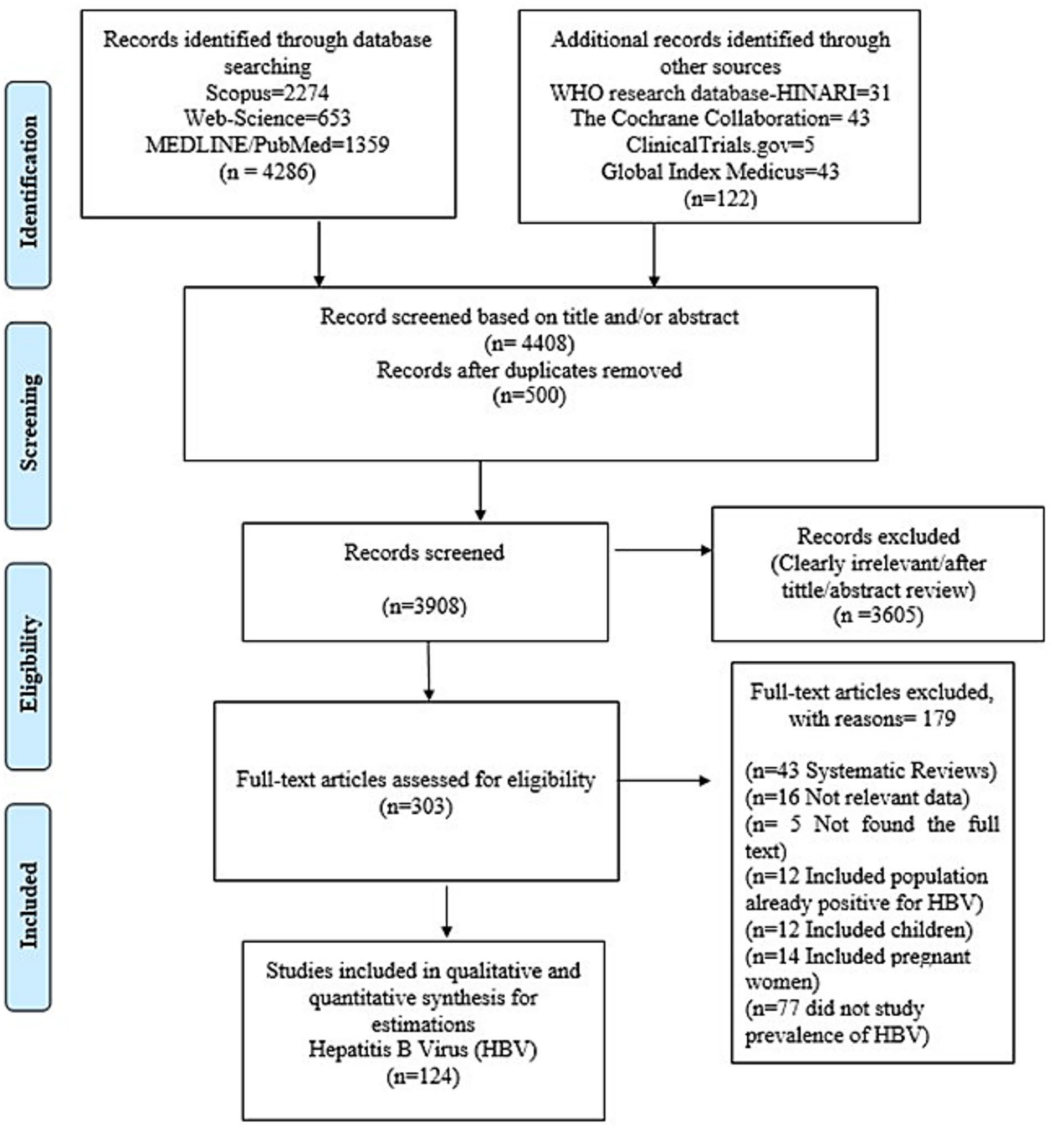
PRISMA flow diagram of studies reviewed, screened, and included.

Among 179 that did not meet our inclusion criteria, 77 did not study the prevalence of Hepatitis B among blood donors, 43 were systematic reviews, 16 studies did not have relevant data, five studies did not have their full text available, 12 studies included population already positive to Hepatitis B, 12 studies included children, 14 studies included pregnant women (See Figure 1; Supplementary Table S3).

### Study characteristics

Supplementary Table S2 shows the characteristics of the studies included in this work. Thirty (55.5%) of the 54 African countries are represented in the 124 studies included. Most of the studies were conducted in Western Africa 51 (41.13%), followed by Eastern Africa 32 (25.81%), then by Central 26 (20.97%), and lastly by the Northern 9 (7.26%) and Southern 6 (4.84%) African region.

The year of study publication ranged from 1990 to 2024. The majority, 89 (75%), were published after 2010. The median proportion of men in the included studies was 83.75%.

Regarding the risk of bias, most studies had a moderate risk of bias 70 (56.45%), followed by a low risk of bias 36 (29.03%), and lastly by a high risk of bias 18 (14.52%).

### Seroprevalence of hepatitis B surface antigen

**Figure 2:**
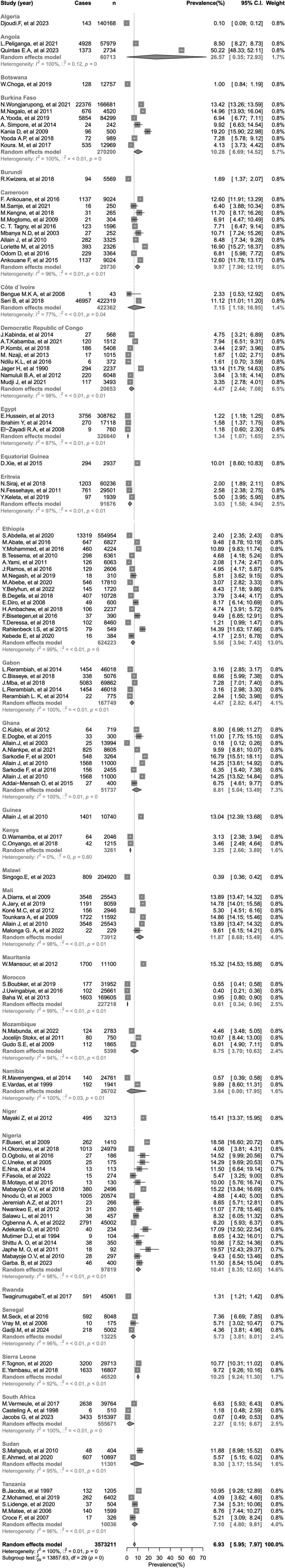
Forest plot of the pooled seroprevalence of Hepatitis B Surface Antigen among Blood donors in Africa by country, Random-effect model: subgroup analysis by region. ES estimated prevalence of HBV.

We found that the pooled seroprevalence of HBsAg among blood donors in Africa was 6.93% (95% CI: 5.95–7.97%; see the forest plot in Figure 2).

**Figure 3:**
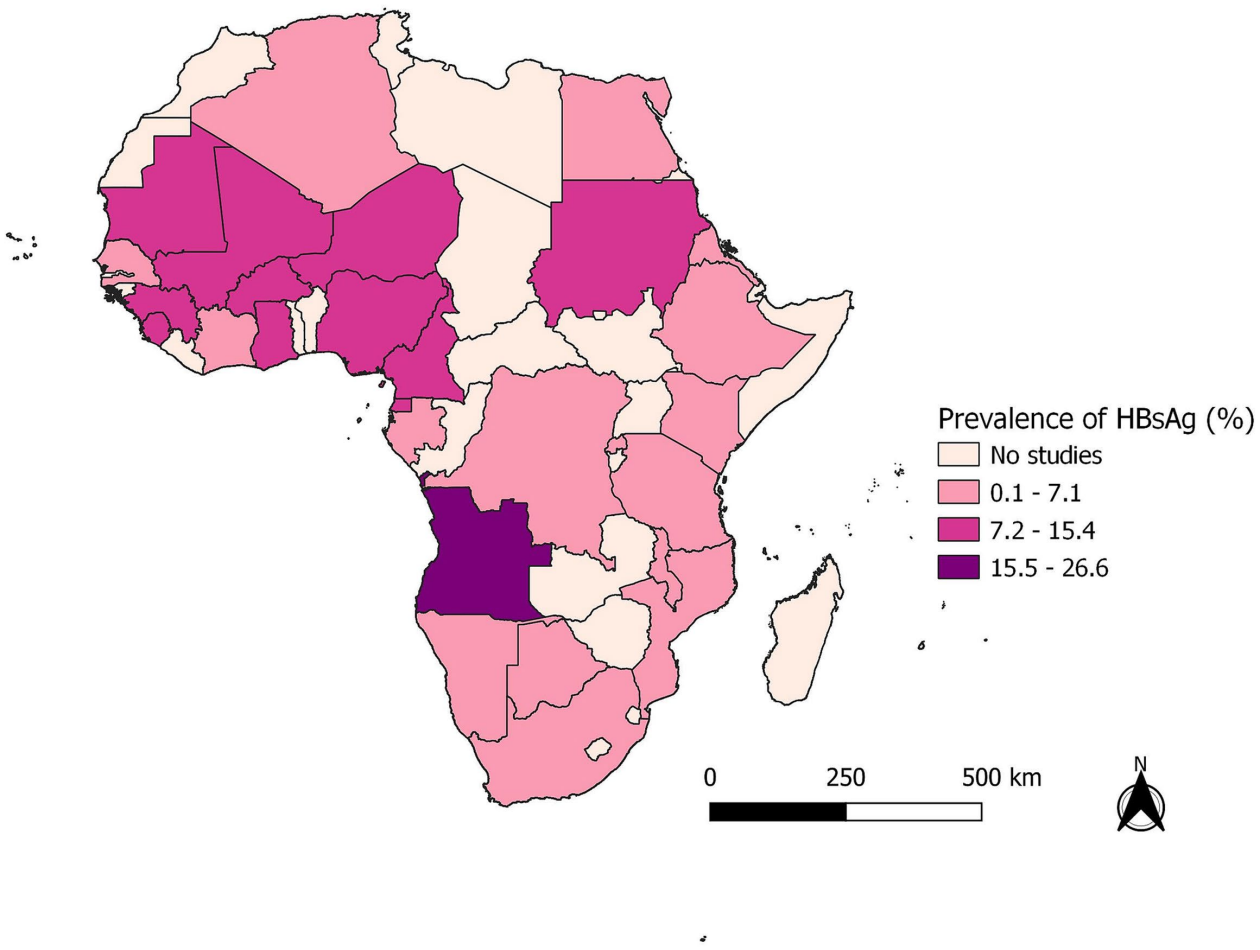
Map of the seroprevalence of Hepatitis B among blood donors in Africa.

In subgroup analysis, we found statistically significant differences in the seroprevalence of HBsAg among blood donors in Africa according to the study country (*p* < 0.01), year of study publication (*p* < 0.03) and African region (*p* < 0.01), (Figures 2, 3; Table 1).

**Table 1:** Sub-group analysis of the pooled prevalence of HBsAg estimation in African blood donors by regions (1990–2024).

Moderator variables	Category	Number of studies	Aggregate sample size	Prevalence % (95% CI)	*I*^2^ (%)	*p*-value
Africa region	Western	51	1,000,828	10.09 (8.75; 11.50)	99.7	0.01
	Eastern	32	990,144	4.87 (3.77; 6.11)	99.7	
	Central	26	281,782	7.81 (5.34; 10.71)	99.7	
	Northern	9	705,327	1.73 (0.45; 3.79)	99.8	
	Southern	6	595,130	2.47 (0.54; 5.75)	99.9	
Year of publication	1990–2000	5	5,997	8.07 (3.80; 13.73)	96.4	0.03
	2001–2010	26	151,880	9.41 (7.19; 11.90)	99.7	
	2011–2024	93	3,415,334	6.26 (5.19; 7.42)	99.9	

*I2 = Heterogeneity; p-value: significance test of subgroup differences.*

Regarding the seroprevalence of HBsAg by African regions, we found that the Western region had the highest prevalence of HBsAg at 10.09% (95% CI: 8.75–11.50%), followed by the Central region with 7.81% (95% CI: 5.34–10.71%), then by the Eastern Africa region with 4.87% (95% CI: 3.77–6.11%) the Southern with 2.47% (95% CI: 0.54–5.75%) and finally, by the Northern African region with 1.73% (95% CI: 0.45–3.79%).

Regarding to the year of study publication, highest prevalence was observed in studies published between 2001 and 2010 (9.41%; 95% CI: 7.19–11.90%) followed by studies published from 1990 to 2000 (8.07%; 95% CI: 3.80–13.73%) and the lowest prevalence was observed in the studies published between 2011 and 2024 (6.26%; 95% CI: 5.19–7.42%) (see Table 1).

We generally found high heterogeneity among pooled studies (Cochran *Q-*test *p* < 0.001 and I^2^ = 100%). In the meta-regression analysis, we observed that the heterogeneity was moderated by the African region (*p* < 0.01) and the country where the study was performed (*p* < 0.01) (see Table 2).

**Table 2:** Moderators of heterogeneity on the seroprevalence of HBsAg in blood donors in Africa.

Variables	Moderators test *p*-value	*R*^2^ (%)
African region	< 0.01	28.60
Country	< 0.01	44.69
Risk of bias	0.92	0.0
Location	0.05	2.05
Setting	0.69	0.00
Type of Blood donors	0.64	0.00
Age	0.89	0.00
Proportion of male	0.31	0.07
Year of study publication	0.07	2.02

*R2: The amount of heterogeneity accounted for.*

Among the studied moderator variables, 44.69% of the heterogeneity was explained by the country where the study was performed (*p* < 0.01), and by the African region 28.60% (*p* < 0.01). We did not find a statistically significant variation in the seroprevalence of HBsAg by the risk of bias (*p* = 0.92), study location (*p* = 0.05), setting (*p* < 0.69), year of study publication (*p* = 0.07) type of blood donor (*p* = 0.64), age (*p* = 0.89) and proportion of males (*p* = 0.31) (see Table 2).

**Figure 4:**
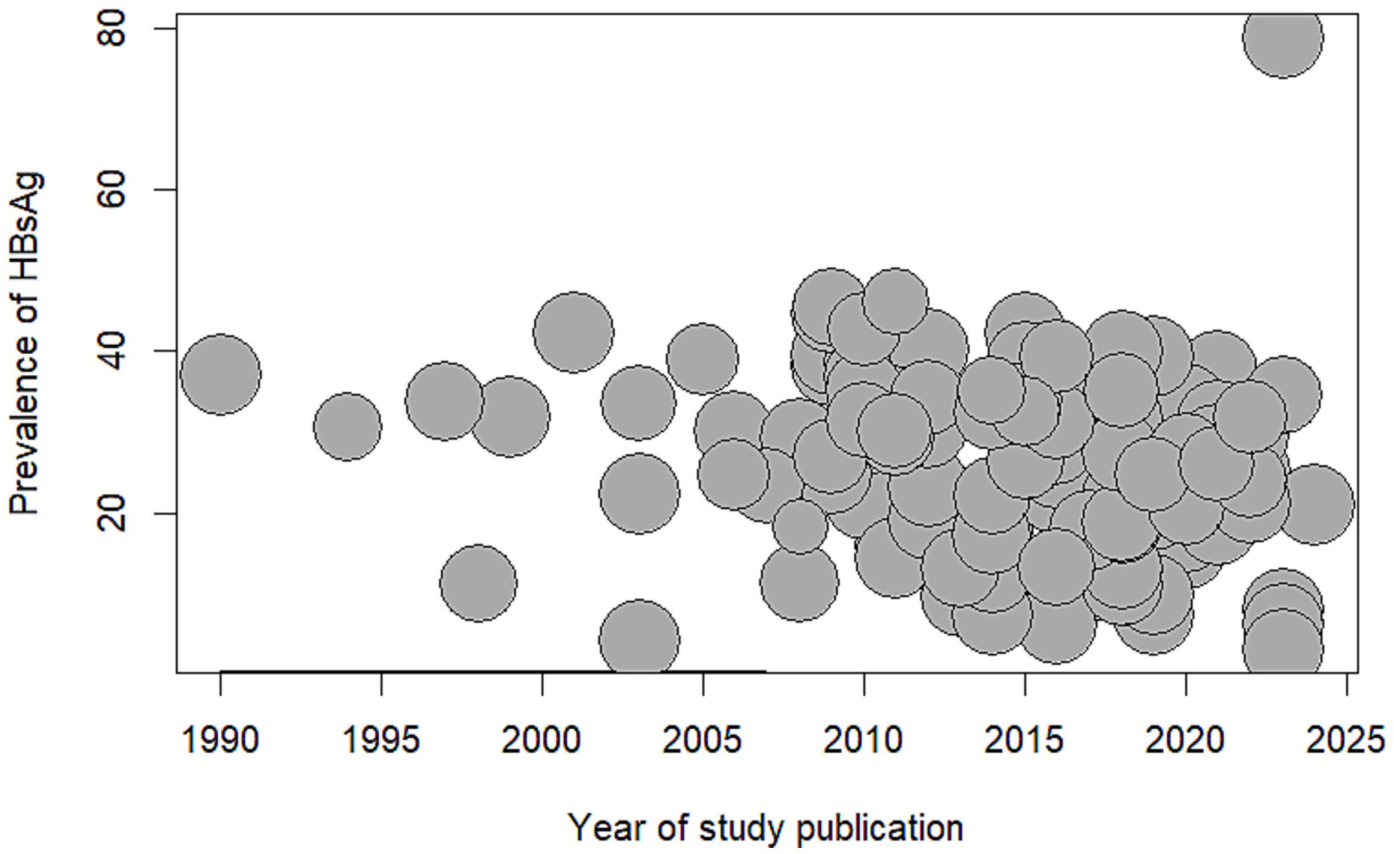
Bubble plot meta-regression of seroprevalence of Hepatitis B Surface Antigen among blood donors in Africa and year of study publication. HBsAg: Hepatitis B Surface Antigen.

Although the year of study publication was not statistically significant in the meta-regression analysis, we did find a decreased trend in the seroprevalence of hepatitis B among African blood donors over the years (see Figure 4).

**Figure 5:**
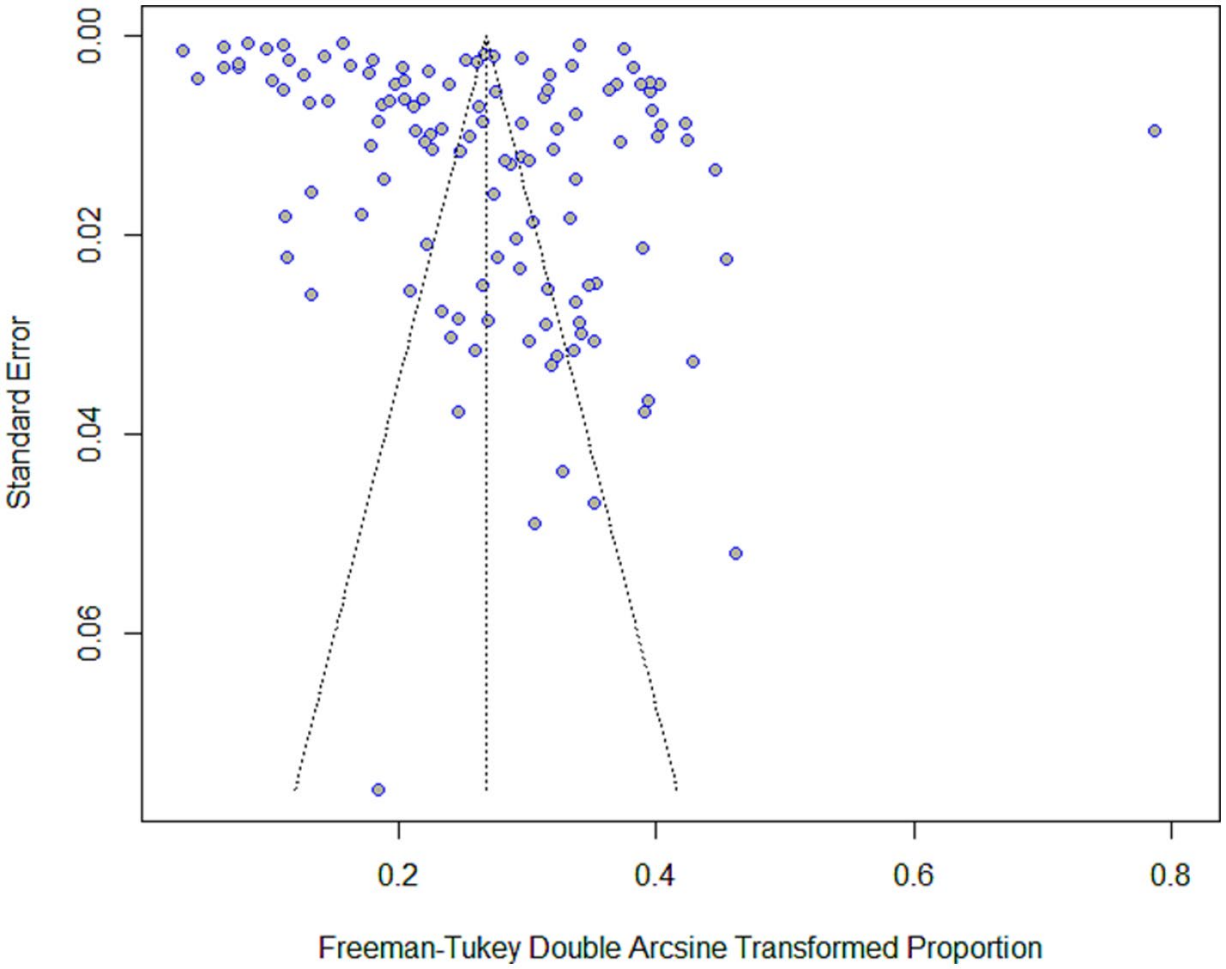
Funnel plot of the seroprevalence of HBV in African blood donors from 1990 to 2022.

The funnel plot showed asymmetry, and the regression Egger’s test was statistically significant (*p* < 0.01). Meaning that the evidence of the presence of risk of publication bias was identified (see Figure 5).

## Discussion

Our study shows that the seroprevalence of HBsAg among blood donors in African countries was 6.93% (95% CI: 5.95–7.97%). This finding is consistent with a report on the prevalence of HBsAg in the general population in Africa, which is considered to be higher (19). This means the Hepatitis B virus remains an enormous public health problem in Africa (5). These findings are worrisome as there are reports of transmission of the Hepatitis B virus infection by blood transfusion (20, 21). The risk of becoming infected with HBV in sub-Saharan Africa from a blood transfusion is high and around 4.3 per 1,000 units (4).

In our study, the seroprevalence of HBsAg among blood donors was higher compared to data reported from the European Union, which is 1.1% among first-time blood donors (22), China 1.32% (23), Laos (Southeast Asian country) which was around 2.6% (24) and in the Eastern Mediterranean and Middle Eastern countries which were 2.03% (25).

We found statistically significant differences in the prevalence of HBsAg based on the African region where the study was performed. The Western Africa region had the highest prevalence of 10.09%, followed by the Central region (7.81%) and Eastern (4.87%), while the Southern (2.47%) and Northern African regions (1.73%) exhibited lower prevalence.

These findings are consistent with the systematic reviews and meta-analyses conducted in countries of the Western region, such as Burkina Faso (26), Kenya (27) in Eastern Africa, and Cameron (28) in the Central region of Africa, which show a higher prevalence of Hepatitis B ranging from 8 to 12%. In contrast, the low prevalence observed in the countries of Northern and Southern Africa is consistent with the epidemiological study on the prevalence of the Hepatitis B virus in Africa, which shows a low endemicity level (<2%) in the Northern region (19, 29).

We found an inverse relationship between the prevalence of Hepatitis B among blood donors in Africa and the year of the study publication, although it was not statistically significant in the meta regression analysis, we did find statistically differences in subgroup analysis splitting the year into three categories. We found that, published studies (after 2010) tended to present lower seroprevalence of Hepatitis B than studies published before 2010. This finding can be explained by the introduction of universal infant and childhood hepatitis B vaccination programs in 1997 (30) and improved screening and treatment of Hepatitis B.

Additionally, we found that the country where the study was carried out was a statistically significant moderator of the heterogeneity of the seroprevalence of HBsAg. These findings can be explained by the existing differences in the access and quality of screening procedures, the social and demographic profile of each country, lifestyle, prevalence of hepatitis B in the general population, and much more importantly, availability of vaccination and treatment services in these countries (19, 31).

Our systematic review has some limitations: The pooled seroprevalence of HBsAg among blood donors that we found cannot be generalized to the whole of Africa as 24 (44%) of African countries did not have any study on the topic. The studies overrepresented countries located in the Western, Central and Eastern regions of Africa and underrepresented those countries in the Northern and Southern regions of the continent. Therefore, further studies are needed concerning underrepresented African areas to complement our findings and to have a good overview of the seroprevalence of HBsAg in Africa. Additionally, we found higher heterogeneity among the included studies (*I*^2^ = 100%). Moreover, we found greater variation in the precision of our estimates due to differences in the total sample sizes of studies across different periods and African regions. Specifically, fewer populations were included in studies conducted in the 1990s compared to the larger number included in studies after 2001. Similarly, smaller sample sizes were observed in the Southern and Northern regions compared to the Western, Eastern, and Central African regions.

Notwithstanding the above limitations, this study has some strengths worth mentioning: to the best of our knowledge, this is the first systematic review and meta-analysis study that analyzed and synthesized the seroprevalence of HBsAg among blood donors in Africa and investigated the reasons for the variability of the prevalence of HBsAg across Africa.

Conclusion: The prevalence of HBsAg among blood donors in Africa is still very high, and it widely varies according to the country, African regions, and year of study publication. Therefore, there is a need for scale-up strategies to intensify the screening of blood donors and extend access to the Hepatitis B vaccine and improve public policy for blood transfusion safety toward Hepatitis B virus elimination.

## Data Availability

The data that support the findings of this study are available from the corresponding author upon reasonable request.
