# Supplementary material for: Seroprevalence of Hepatitis B virus surface antigen among African blood donors: a systematic review and meta-analysis
Source: Front Public Health. 2024 Oct 21;12:1434816. doi: 10.3389/fpubh.2024.1434816 (PMC11532187; doi:10.3389/fpubh.2024.1434816)
Supplement: Supplementary file 1 [file Table_1.DOCX]

**Supplementary materials**

**Table S1: Research query**

| **Search** | **Query** |
| --- | --- |
| #12 | Search: **#9 OR #4 AND #5 OR #6 AND #7** |
| #11 | Search: **#9 AND #10** |
| #10 | Search: **#4 OR #5 OR #6 OR #7** |
| #9 | Search: **#8 AND #3** |
| #8 | Search: **#1 AND #2** |
| #7 | Search: **((Syphilis OR Lues))** |
| #6 | Search: **((Hepaciviruses OR (Hepatitis C Virus OR Hepatitis C viruses) OR (Hepatitis C-Like Virus OR Hepatitis C-Like Viruses))** |
| #5 | Search: **((Hepatitis B virus OR Hepatitis B viruses) OR (Dane Particle OR Particle Dane) OR (Hepatitis Virus OR Homologous Serum))** |
| #4 | Search: **((HIV OR Human Immunodeficiency Virus OR Human Immunodeficiency Viruses) OR (AIDS Virus OR AIDS Viruses) OR (Acquired Immune Deficiency Syndrome Virus OR Acquired Immunodeficiency Syndrome Virus) OR (Human T Lymphotropic Virus Type III OR Human T-Lymphotropic Virus Type III) OR (Lymphadenopathy-Associated Virus OR Lymphadenopathy Associated Virus OR Lymphadenopathy-Associated Viruses) OR (HTLV-III OR LAV-HTLV-III))** |
| #3 | Search: **Algeria OR Angola OR ((Benin OR (Republic of Benin) OR Dahomey)) OR (Botswana OR Bechuanaland OR Kalahari) OR ((Burkina Faso OR (Upper Volta) OR (Burkina Fasso)) OR ((Burundi OR (Republic of Burundi) OR Urundi)) OR ((Cabo Verde) OR (Republic of Cape Verde) OR (Cape Verde)) OR ((Cameroon OR (Republic of Cameron) OR (United Republic of Cameroon) OR Cameroons)) OR ((Central African Republic) OR Ubangi-Shari) OR Chad OR ((Comoros OR (Iles Comores) OR (Comoro Islands) OR Mayotte)) OR ((Democratic Republic of Congo) OR Congo OR (Kinshasa) OR Zaire OR (Belgian Congo) OR Katanga)) OR ((Republic of Congo OR Republic of the Congo OR Congo (Brazzaville)) OR ((Cote d'Ivoire OR (Ivory Coast) OR (Republic of Cote diIvoire)) OR ((Djibouti OR (Republic of Djibouti) OR (French Somaliland)) OR ((Egypt OR (Arab Republic of Egypt) OR (United Arab Republic)) OR ((Equatorial Guinea OR (Republic of Equatorial Guinea) OR (Spanish Guinea) OR (Guinea Spanish) OR (Rio Muni)) OR Eritrea OR (Eswatini OR Swaziland) OR ((Ethiopia OR (Federal Democratic Republic of Ethiopia)) OR ((Gabon OR (Gabonese Republic)) OR ((Gambia OR (Republic of the Gambia)) OR ((Ghana OR (Republic of Ghana) OR (Gold Coast)) OR ((Guinea OR (Republic of Guinea) OR (French Guinea)) OR ((Guinea-Bissau OR (Republic of Guinea-Bissau) OR (Portuguese Guinea)) OR ((Kenya OR (Republic of Kenya)) OR ((Lesotho OR Basutoland OR (Kingdom of Lesotho)) OR ((Liberia OR (Republic of Liberia)) OR Libya OR ((Madagascar OR (Malagasy Republic)) OR ((Malawi OR (Republic of Malawi) OR Nyasaland) OR ((Mali OR (Republic of Mali)) OR Mauritania OR ((Mauritius OR (Agalega Islands)) OR (Morocco OR Ifni) OR ((Mozambique OR (Republic of Mozambique) OR Mosambique OR Mocambique OR Moçambique OR (Portuguese East Africa)) OR ((Namibia OR (Southwest Africa) OR (Republic of Namibia) OR (South West Africa)) OR ((Niger OR (Republic of Niger)) OR ((Nigeria OR (Federal Republic of Nigeria)) OR ((Rwanda OR (Republic of Rwanda)) OR (Sao Tome and Principe) OR ((Senegal OR (Republic of Senegal)) OR Seychelles OR ((Sierra Leone) OR (Republic of Sierra Leone)) OR Somalia OR ((South Africa) OR (Union of South Africa) OR (Republic of South Africa)) OR (South Sudan) OR ((Sudan OR (Republic of the Sudan)) OR ((Tanzania OR (United Republic of Tanzania) OR Zanzibar OR Tanganyika)) OR ((Togo OR (Togolese Republic)) OR Tunisia OR ((Uganda OR (Republic of Uganda)) OR ((Zambia OR (Northern Rhodesia) OR (Republic of Zambia)) OR ((Zimbabwe OR (Zimbabwe Rhodesia) OR (Southern Rhodesia) OR (Republic of Zimbabwe))** |
| #2 | Search: **((Prevalence OR Prevalences) OR (Seroprevalence OR Seroprevalences) OR (Seroepidemiologic OR Seroepidemiological))** |
| #1 | Search: **Blood AND ((Donor OR Donors) OR (Donation OR Donations))** |
