# Supplementary material for: Seroprevalence of Hepatitis B virus surface antigen among African blood donors: a systematic review and meta-analysis
Source: Front Public Health. 2024 Oct 21;12:1434816. doi: 10.3389/fpubh.2024.1434816 (PMC11532187; doi:10.3389/fpubh.2024.1434816)
Supplement: Supplementary file 2 [file Table_2.DOCX]

**TABLE-S2**. Characteristics of all Studies included in the systematic review and meta-analysis of TTIs (Seroprevalence of Hepatitis B Virus) among African Blood Donors

| **First Author**  **and colleagues** | **Year of Publication** | **Study Design** | **Country** | **Enrolment time** | **Sample Size** | **Total participants in Study (N)** | **Blood donors by sex Male N(%)** | **Blood donors type VNRBD (N)** | **Blood donors RD-Paid (N)** | **Family donors (FRD) (N)** | **HBV (HBsAg) Diagnosis/Screening method** | **HBsAg overall positivity (N)** | **HBsAg overall positivity rates (%)** | **Risk of**  **Bias** |
| --- | --- | --- | --- | --- | --- | --- | --- | --- | --- | --- | --- | --- | --- | --- |
| Siraj N. et al. | 2018 | Retrospective | Eritreia | 2010-2016 | 60236 | 60236 | 39978 (66.4) | 54264 | 5972 | - | 3rd Gen ELISA | 1203 | 2.0 | ***Low*** |
| Abdella S. et al. | 2020 | Retrospective | Ethiopia | 2014-2019 | 554954 | 554954 | 354707 (63.9) | 520658 | 34296 | - | 3rd Gen ELISA | 13319 | 2.4 | ***Low*** |
| Buseri F. et al. | 2009 | Prospective | Nigeria | 2007-2008 | 1410 | 1410 | 1200 (85.1) | - | - | - | ELISA | 262 | 18.6 | ***Moderate*** |
| Okoroiwu H. et al. | 2018 | Both | Nigeria | 2005-2016 | 24979 | 24979 | 24654 (98.6) | 137 | 15487 | 9355 | Immunochromatography | 1013 | 4.1 | ***Low*** |
| Fessehaye N. et al. | 2011 | Retrospective | Eritrea | 2006-2009 | 29501 | 29501 | - | 23385 | 6116 | 6116 | - | 761 | 2.58 | ***Moderate*** |
| Nzaji M. et al. | 2013 | Retrospective | Democratic Republic of Congo | 2008 | 1015 | 1015 | 965 (95.1) | 493 | 522 | - | Determine™ HBSAg | 17 | 1.6 | ***Moderate*** |
| Deressa T. et al. | 2018 | Retrospective | Ethiopia | 2014-2017 | 8460 | 8460 | 5644 (66.7) | - | - | - | Hepanostika HBsAg Uni-form II | 102 | 1.2 | ***Moderate*** |
| Diarra A. et al. | 2009 | Retrospective | Mali | 2007 | 25543 | 25543 | - | 8094 | 17449 | - | Monolisa AgHBS | 3548 | 13.9 | ***Moderate*** |
| Stokx J. et al. | 2011 | Retrospective | Mozambique | 2009 | 750 | 750 | - | - | - | - | RDTs | 80 | 10.6 | ***Moderate*** |
| Ankouane F. et al. | 2016 | Retrospective | Cameroon | 2013 | 9024 | 9024 | 8453 (93.6) | 249 | 8767 | - | ELISA | 1137 | 12.6 | ***Moderate*** |
| Abate M. et al. | 2016 | Retrospective | Ethiopia | 2010-2014 | 6827 | 6827 | 6648 (97.3) | - | - | - | ELISA | 647 | 9.48 | ***Moderate*** |
| Mohammed Y. et al. | 2016 | Retrospective | Ethiopia | 2010-2013 | 4224 | 4224 | 4171 (98.7) | 85 | 4139 | - | ELISA | 460 | 10.89 | ***Moderate*** |
| Tessema B. et al. | 2010 | Retrospective | Ethiopia | 2003-2007 | 6361 | 6361 | 5592 (87.9) | - | - | - | Hepanostika HBsAg Ultra | 298 | 4.7 | ***Moderate*** |
| Kubio C. et al. | 2012 | Retrospective | Ghana | 2009 | 843 | 719 | - | - | 201 | 518 | HBsAg Acon | 64/843 | 7.5 | ***Moderate*** |
| Mavenyengwa R. et al. | 2014 | Retrospective | Namibia | 2012 | 24761 | 24761 | 13054 (52.7) | - | - | - | NAT | 140 | 0.6 | ***High*** |
| Keleta Y. et al. | 2019 | Retrospective | Eritrea | 2014-2017 | 1939 | 1939 | 1710 (88.2) | 781 | 1158 | 1158 | ELISA | 97 | 5 | ***Moderate*** |
| Wongjarupong N. et al | 2021 | Retrospective | Burkina Faso | 2009-2013 | 166681 | 166681 | 119437 (71.7) | - | - | - | Hepanostika HBsAg Ultra | 22376 | 13.4 | ***Moderate*** |
| Nagalo M. et al. | 2011 | Retrospective | Burkina Faso | 2009 | 4520 | 4520 | 3418 (75.6) | - | - | - | Hepanostika HBsAg Ultra | 676 | 14.96 | ***Low*** |
| Peliganga L. et al. | 2021 | Retrospective | Angola | 2005-2020 | 57979 | 57979 | 41414 (71.4) | - | - | - | RDTs | 4928 | 8.5 | ***Low*** |
| Kabinda J. et al. | 2014 | Retrospective | Democratic Republic of Congo | 2011 | 593 | 568 | 417 (73.4) | 513 | 4 | 60 | 2™ Determine HBsAg | 27 | 4.8 | ***High*** |
| Rerambiah L. et al. | 2014 | Retrospective | Gabon | 2009-2011 | 46018 | 46018 | 31846 (69.2) | 19378 | 21696 | - | HBsAg ultra from Bio-rad | 1454 | 3.16 | ***Moderate*** |
| Yami A. et al. | 2011 | Retrospective | Ethiopia | 2010 | 9204 | 6063 | 4802 (79.2) | - | - | - | ELISA | 126 | 2.08 | ***Moderate*** |
| Mahgoub S. et al. | 2010 | Retrospective | Sudan | 2010 | 404 | 404 | 403 (99.7) | - | - | - | Enzygnost 5.0 | 48 | 11.8 | ***Moderate*** |
| Ogbolu D, et al. | 2016 | Prospective | Nigeria | - | 186 | 186 | 141 (75.8) | 30 | 3 | 153 | ELISA | 27 | 14.52 | ***High*** |
| Samje M. et al. | 2021 | Retrospective | Cameroon | 2019 | 494 | 250 | 176 (70.4) | 97 | 150 | - | Immunochromatography | 16 | 6.4 | ***Moderate*** |
| Bisseye C. et al. | 2018 | Retrospective | Gabon | 2012-2017 | 5706 | 5076 | 4765 (93.8) | - | 5706 | 5706 | RDTs | 338 | 6.7 | ***Low*** |
| Kengne M. et al. | 2018 | Prospective | Cameroon | 2014 | 265 | 265 | 242 (91.3) | 30 | 235 | 235 | ELISA | 31 | 11.7 | ***High*** |
| Uneke CJ. et al. | 2005 | Retrospective | Nigeria | 1999-2002 | 175 | 175 | - | - | - | - | ELISA | 25 | 14.3 | ***Moderate*** |
| Ramos J. et al. | 2016 | Retrospective | Ethiopia | 2007-2012 | 9493 | 2606 | - | - | - | - | RDTs | 129 | 4.95 | ***High*** |
| Kabamba A. et al. | 2021 | Retrospective | Democratic Republic of Congo | 2017-2019 | 1512 | 1512 | 1081 (71.5) | 394 | 1118 | - | ELISA | 120 | 7.9 | ***Moderate*** |
| Negash M. et al. | 2019 | Retrospective | Ethiopia | 2017-2018 | 338 | 310 | 198 (63.8) | - | - | - | ELISA | 18 | 5.8 | ***Moderate*** |
| Jary A. et al. | 2019 | Retrospective | Mali | 2018 | 8207 | 8059 | 7157 (88.8) | 160 | 7898 | - | ELISA | 1191 | 14.78 | ***Moderate*** |
| Tognon F. et al. | 2020 | Retrospective | Sierra Leone | 2013-2016 | 30467 | 29713 | 22736 (76.5) | 2862 | 23844 | - | RDTs | 3200 | 10.8 | ***Moderate*** |
| Xie D. et al. | 2015 | Retrospective | Equatorial Guinea | 2011-2013 | 2937 | 2937 | 2256 (76.8) | - | - | - | Immunochromatography | 294 | 10.01 | ***High*** |
| Vermeule M. et al. | 2017 | Retrospective | South Africa | 2012-2015 | 3075422 | 397632 | 177729 (44.7) | - | - | - | Hepanostika Ultra | 2638 | 6.63 | ***Moderate*** |
| Kombi P. et al. | 2018 | Retrospective | Democratic Republic of Congo | 2013-2015 | 5408 | 5408 | 5121 (94.7) | 5259 | - | - | RDTs | 186 | 3.5 | ***Moderate*** |
| Abebe M. et al. | 2020 | Retrospective | Ethiopia | 2015-2019 | 17810 | 17810 | 12480 (70.1) | - | - | - | ELISA | 546 | 3.07 | ***Low*** |
| Vardas E. et al. | 1999 | Retrospective | Namibia | 1997 | 1941 | 1941 | 816 (42) | - | - | - | Radioimmunoassay | 192 | 9.89 | ***Low*** |
| Boubker S. et al. | 2019 | Retrospective | Morocco | 2013-2015 | 31952 | 31952 | 23177 (72.5) | - | - | - | ELISA | 177 | 0.55 | ***Moderate*** |
| Nna E. et al. | 2014 | Retrospective | Nigeria | 2014 | 113 | 113 | - | - | - | - | RDTs | 13 | 11.5 | ***Moderate*** |
| Uwingabiye J. et al. | 2016 | Retrospective | Morocco | 2010-2012 | 25661 | 25661 | 24378 (95) | - | - | - | ELISA | 102 | 0.40 | ***High*** |
| Wamamba D. et al. | 2017 | Retrospective | Kenya | 2015 | 3690 | 2046 | 1360 (66.5) | - | - | - | ELISA | 64 | 3.1 | ***Low*** |
| Dogbe E. et al. | 2015 | Retrospective | Ghana | - | 300 | 300 | - | - | - | - | 3rd Gen ELISA | 33 | 11 | ***Moderate*** |
| Kwizera R. et al. | 2018 | Retrospective | Burundi | 2016 | 8993 | 5569 | 2660 (48) | - | - | - | ELISA | 94 | 1.7 | ***Moderate*** |
| Mabunda N. et al. | 2022 | Prospective | Mozambique | 2014-2015 | 2783 | 2783 | 2320 (83.3) | 1146 | 1608 | - | RDTs | 124 | 4.5 | ***Moderate*** |
| Fasola F. et al. | 2022 | Retrospective | Nigeria | 2019-2020 | 274 | 274 | 237 (86.4) | - | - | - | RDTs | 15 | 5.5 | ***Low*** |
| Mudji J. et al. | 2021 | Retrospective | Democratic Republic of Congo | 2016-2018 | 3497 | 3497 | 3232 (92.4) | 492 | 70 | 2931 | RDTs | 117 | 3.4 | ***Low*** |
| Choga W. et al. | 2019 | Prospective | Botswana | 2014-2015 | 12575 | 12757 | 8513 (66.7) | - | - | - | ELISA | 128 | 1.02 | ***Low*** |
| Mansour W. et al. | 2012 | Prospective | Mauritania | 2008-2009 | 11100 | 11100 | - | - | - | - | ELISA | 1700 | 15.3 | ***Low*** |
| Twagirumugabe T. et al. | 2017 | Retrospective | Rwanda | 2017 | 45061 | 45061 | 33875 (75) | - | - | - | ELISA | 591 | 1.26 | ***Moderate*** |
| Mba J. et al. | 2018 | Retrospective | Gabon | 2009-2016 | 69862 | 69862 | 53390 (74.4) | 25594 | - | 44268 | 4th ELISA | 5083 | 7.28 | ***Low*** |
| Yooda A. et al. | 2019 | Retrospective | Burkina Faso | 2015-2017 | 84299 | 84299 | 59979 (71.1) | - | - | - | Architect HBsAg Qualitative II | 5854 | 6.94 | ***Moderate*** |
| Allain J. et al. | 2003 | Retrospective | Ghana | 1999-2000 | 13994 | 13994 | - | 6538 | 7456 | - | EIA | 25 | 0.18 | ***Low*** |
| Nlankpe A. et al. | 2021 | Retrospective | Ghana | 2013-2017 | 8605 | 8605 | 8517 (98.9) | - | - | - | DiaSpot | 825 | 9.59 | ***Low*** |
| Jacobs B. et al. | 1997 | Retrospective | Tanzania | 1992 | 2333 | 1205 | 1074 (89.1) | - | - | - | ELISA | 132 | 10.95 | ***Moderate*** |
| Mohamed Z. et al. | 2019 | Retrospective | Tanzania | 2016-2017 | 6402 | 6402 | 5383 (84) | 763 | 763 | 5634 | ELISA | 262 | 4.1 | ***High*** |
| Belyhun Y. et al. | 2022 | Retrospective | Ethiopia | 2013 | 4105 | 1720 | 64 (3.7) | - | - | - | RDTs | 145 | 8.4 | ***Moderate*** |
| Hussein E. et al. | 2014 | Retrospective | Egypt | 2006-2012 | 308762 | 308762 | - | 195635 | - | 113127 | ELISA | 3756 | 1.22 | ***Moderate*** |
| Degefa B. et al. | 2018 | Retrospective | Ethiopia | 2011-2014 | 10728 | 10728 | 3750 (35) | 6302 | 4426 | - | ELISA | 407 | 3.79 | ***Moderate*** |
| Koné M.C. et al. | 2012 | Retrospective | Mali | 2007-2010 | 2946 | 2946 | - | 27 | 121 | 121 | Test Hepa-Scan | 156 | 5.3 | ***Moderate*** |
| Mogtomo M. et al. | 2009 | Prospective | Cameroon | 1995-2004 | 1513 | 304/1513 | 1171 (77.3) | 80 | 1433 | 1433 | Radioimmunoassay | 21 | 6.91 | ***High*** |
| Tounkara A. et al. | 2009 | Retrospective | Mali | 2001-2002 | 11592 | 11592 | 10108 (87.1) | - | - | - | ELISA | 1722 | 14.9 | ***High*** |
| Diro E. et al. | 2008 | Retrospective | Ethiopia | 2003-2004 | 1761 | 600 | 537 (89.5) | - | - | - | ELISA | 49 | 8.2 | ***Moderate*** |
| Ambachew H. et al. | 2018 | Retrospective | Ethiopia | 2016 | 2237 | 2237 | - | - | - | - | ELISA | 106 | 4.7 | ***Low*** |
| Motayo B. et al. | 2015 | Prospective | Nigeria | 2013 | 130 | 130 | 126 (96.9) | - | 130 | - | EIA | 13 | 10 | ***Moderate*** |
| Seck M. et al. | 2016 | Prospective | Senegal | - | 8219 | 8048 | 6439 (80) | - | - | - | ELISA | 592 | 7.4 | ***Moderate*** |
| Alain C. et al. | 2020 | Retrospective | Democratic Republic of Congo | 2016-2017 | 360 | 360 | 301 (83.6) | - | - | - | - | 38 | 10.6 | ***Low*** |
| Lidenge S. et al. | 2020 | Retrospective | Tanzania | 2019 | 504 | 504 | 431 (85.5) | - | - | - | 3rd Gen ELISA | 37 | 7.3 | ***Low*** |
| Yambasu E. et al. | 2018 | Retrospective | Sierra Leone | 2016 | 16865 | 16807 | 13426 (79.8) | 1986 | - | 14760 | Rapid diagnostic test (RDT) | 1633 | 9.7 | ***High*** |
| Simpore A. et al. | 2014 | Retrospective | Burkina Faso | 2011-2012 | 6375 | 242 | - | - | - | - | ELISA | 24 | 9.9 | ***Moderate*** |
| Tagny C. et al. | 2016 | Retrospective | Cameroon | 2011-2015 | 1704 | 1596 | 1313 (82.2) | 403 | 1193 | 1193 | 3rd Gen ELISA | 123 | 7.7 | ***Moderate*** |
| Ahmed E. et al. | 2020 | Retrospective | Sudan | 2017 | 10897 | 10897 | 10897 (100-all men) | 10897 | - | - | Immunochromatography | 607 | 5.57 | ***Moderate*** |
| Onyango C. et al. | 2018 | Retrospective | Kenya | 2015-2016 | 1215 | 1215 | 700 (57.6) | - | - | - | ELISA | 42 | 3.46 | ***Moderate*** |
| Bisetegen F. et al. | 2016 | Retrospective | Ethiopia | 2015 | 390 | 390 | 291 (74.6) | - | - | - | ELISA | 37 | 9.5 | ***High*** |
| Ibrahim Y. et al. | 2014 | Retrospective | Egypt | 2010-2011 | 17118 | 17118 | 13918 (81.3) | 2101 | 15017 | 15017 | HBsAg | 270 | 2 | ***Moderate*** |
| Matee M. et al. | 2006 | Retrospective | Tanzania | 2005 | 1599 | 1599 | 1424 (89) | 474 | 1125 | - | 3rd Gen ELISA | 140 | 8.8 | ***Moderate*** |
| Mabayoje O.V. et al. | 2018 | Prospective | Nigeria | 2004-2005 | 2496 | 2496 | 1988 (79.6) | VNRBD | RD | - | ELISA | 380 | 15.22 | ***Low*** |
| Rahlenbeck I S. et al. | 2015 | Retrospective | Ethiopia | 1994-1995 | 2186 | 549 | - | VNRBD | - | - | 3rd Gen ELISA | 79 | 14.4 | ***Low*** |
| Nnodu O. et al.. | 2003 | Prospective | Nigeria | 1990-2002 | 20574 | 20574 | - | VNRBD | - | - | ELISA | 1005 | 4.8 | ***Moderate*** |
| Jeremiah A.Z. et al. | 2011 | Retrospective | Nigeria | 2010-2011 | 266 | 266 | 244 (91.7) | VNRBD | - | - | ELISA | 23 | 8.6 | ***Moderate*** |
| Nwankwo E. et al. | 2012 | Retrospective | Nigeria | 2008 | 280 | 280 | 276 (98.5) | 61 | 62 | 157 | HBsAg Kit | 31 | 11.1 | ***High*** |
| El-Zayadi R.A. et al. | 2008 | Retrospective | Egypt | 2005 | 760 | 760 | 636 (83.6) | VNRBD | - | - | EIA | 9 | 1.18 | ***Moderate*** |
| Ndilu K.L. et al. | 2016 | Retrospective | Democratic Republic of Congo | 2012-2013 | 372 | 372 | 252 (67.7) | VNRBD | - | - | - | 6 | 1.6 | ***High*** |
| Kania D. et al. | 2009 | Retrospective | Burkina Faso | 2002 | 500 | 500 | - | 500 | - | - | 4th Gen ELISA | 96 | 19.2 | ***Moderate*** |
| Vray M. et al. | 2006 | Retrospective | Senegal | 2003 | 290 | 175 | 135 (77.1) | VNRBD | - | - | EIA | 10 | 5.71 | ***Moderate*** |
| Mbanya N.D. et al. | 2003 | Retrospective | Cameroon | 2001 | 264 | 252 | 197 (78.1) | VNRBD | - | - | HBsAntigen Slide ™ | 27 | 10.7 | ***High*** |
| Salawu L. et al. | 2011 | Retrospective | Nigeria | - | 495 | 457 | 443 (96.9) | VNRBD | - | - | ELISA | 38 | 8.32 | ***Moderate*** |
| Casteling A. et al. | 1998 | Retrospective | South Africa | - | 532 | 510 | 275 (53.9) | VNRBD | - | FRD | ELISA | 6/510 | 1.2 | ***Moderate*** |
| Gudo S.E. et al. | 2009 | Retrospective | Mozambique | 2006 | 2019 | 1865 | - | VNRBD | - | - | EIA | 112 | 7.30 | ***Moderate*** |
| Bengue M.K A. et al. | 2008 | Retrospective | Côte d'Ivoire | 2008 | 2866 | 2866 | 35 (1.2) | VNRBD | - | - | EIA | 1 | 2.3 | ***Moderate*** |
| Sarkodie F. et al. | 2001 | Retrospective | Ghana | 1999 | 3264 | 3264 | - | 1492 | 1772 | - | EIA | 548 | 17 | ***Moderate*** |
| Allain J. et al. | 2010 | Retrospective | Mali | 2010 | 25543 | 25543 | - | 8094 | 17449 | - | - | 3548 | 13.9 | ***Low*** |
| Allain J. et al. | 2010 | Retrospective | Cameroon | 2010 | 3325 | 3325 | - | 272 | 3053 | - | - | 282 | 8.48 | ***Low*** |
| Allain J. et al. | 2010 | Retrospective | Ghana | 2010 | 11000 | 11000 | - | 6640 | 4360 | - | - | 1568 | 14.3 | ***Low*** |
| Allain J. et al. | 2010 | Retrospective | Guinea | 2010 | 10740 | 10740 | - | 1784 | 8956 | - | - | 1401 | 13.04 | ***Low*** |
| Sarkodie F. et al. | 2016 | Retrospective | Ghana | 2014 | 2455 | 2455 | 1959 (79.7) | 1080 | - | 1133 | - | 156 | 6.4 | ***Low*** |
| Allain J. et al. | 2010 | Retrospective | Ghana | 2008 | 11000 | 11000 | 7901 (71.8) | 6640 | 4360 | - | DetermineTM HBS Ag | 1568 | 14.3 | ***Low*** |
| Loriette M. et al. | 2015 | Retrospective | Cameroon | 2013 | 2 326 | 2 326 | - | - | - | - | Vikia HBsAg | 393 | 16.9 | ***Moderate*** |
| Malonga G.A. et al. | 2022 | Retrospective | Mali | 2019-2020 | 229 | 229 | - | - | - | - | - | 22 | 9.6 | ***Moderate*** |
| Jager H. et al. | 1990 | Retrospective | Democratic Republic of Congo | 1989 | 2237 | 2237 | 2031 (90.7) | 275 | 571 | 1391 | ELISA | 294 | 13.1 |  |
| Dionne-Odom J. et al. | 2016 | Retrospective | Cameroon | 2014 | 3364 | 3364 | - | 3364 | - | - | RDTs | 229 | 6.8 | **Low** |
| Aluora O.P. et al. | 2020 | Retrospective | Kenya | - | 300 | 300 | - | - | - | - | ELISA | 7 | 2.3 | ***Moderate*** |
| Ogbenna A.A. et al. | 2022 | Retrospective | Nigeria | 2015-2019 | 45 002 | 45 002 | - | - | - | - | ELISA | 2791 | 6.2 | ***Moderate*** |
| Adekanle O | 2010 | Prospective | Nigeria | 2008-2009 | 234 | 234 | 223 (95.2) | - | - | - | ELISA | 40 | 17.1 | ***Moderate*** |
| Croce F. et al. | 2007 | Retrospective | Tanzania | 2002 | 326 | 326 | 266 (81.5) | - | - | - | ELISA | 17 | 5.2 | ***Moderate*** |
| Mutimer J.D. et al. | 1994 | Retrospective | Nigeria | - | 104 | 104 | - | - | 104 | - | EIA | 9 | 8.7 | ***High*** |
| Seri B. et al. | 2018 | Retrospective | Côte d'Ivoire | 1992-2012 | 422 319 | 422 319 | 312516 (73.9) | 422319 | - | - | ELISA | 46957 | 11.11 | ***Moderate*** |
| Ankouane F. et al. | 2015 | Retrospective | Cameroon | 2013 | 9024 | 9024 | 8453 (93.6) | 249 | - | 8767 | ELISA | 1137 | 12.6 | ***Moderate*** |
| Kebede E. et al. | 2020 | Retrospective | Ethiopia | 2018 | 384 | 384 | 213 (55.4) | 384 | - | - | ELISA | 16 | 4.2 | ***Moderate*** |
| Baha W. et al. | 2013 | Retrospective | Morocco | 2008-2010 | 169605 | 169605 | 135175 (79.6) | 169605 | - | - | ELISA | 1603 | 0.96 | ***Moderate*** |
| Addai-Mensah O. et al. | 2015 | Retrospective | Ghana | 2014 | 400 | 400 | 356 (89) | 200 | - | 200 | RDTs | 27 | 6.8 | ***Moderate*** |
| Mayaki Z. et al. | 2012 | Retrospective | Niger | 2010 | 3213 | 3213 | 2574 (80.1) | 2204 | - | 1009 | EIA | 495 | 15.4 | ***Moderate*** |
| Namululi B.A. et al. | 2012 | Retrospective | Democratic Republic of Congo | 2001-2005 | 7 442 | 6 048 | 2301 (38) | 2898 | - | 382 | ELISA | 220/6048 | 3.6 | ***Moderate*** |
| Shittu O.A. et al. | 2014 | Prospective | Nigeria | - | 350 | 350 | 339 (96.8) | - | - | - | RDTs | 38 | 10.9 | ***Moderate*** |
| Mudji J. et al. | 2021 | Retrospective | Democratic Republic of Congo | 2016-2018 | 3493 | 3493 | 3232 (92.5) | - | - | - | RDTs | 117 | 3.4 | ***Moderate*** |
| Japhe O.M. et al. | 2011 | Retrospective | Nigeria | 2009 | 92 | 92 | 69 (67.3) | - | - | - | ELISA | 18 | 19.6 | ***Moderate*** |
| Yooda P.A. et al. | 2018 | Retrospective | Burkina Faso | 2017 | 989 | 989 | 655 (66.2) | - | - | - | ELISA | 72 | 7.28 | ***High*** |
| Rerambiah L. et al.. | 2014 | Retrospective | Gabon | 2009-2011 | 46018 | 46018 | 31846 (69.2) | 19378 | 21696 | - | HBsAg ultra from Bio-rad | 1454 | 3.16 | ***Moderate*** |
| Rerambiah K.L. et al. | 2014 | Retrospective | Gabon | 2014 | 775 | 775 | 552 (71.2) | - | - | - | Monolisa AgHBS | 22 | 2.84 | ***Moderate*** |
| Mabayoje O.V. et al. | 2010 | Retrospective | Nigeria | 2003 | 297 | 297 | 137 (46) | - | - | - | Elisa | 380 | 15.22 | ***High*** |
| Quintas E.A. et al | 2023 | Retrospective | Angola | 2011-2016 | 2734 | 2734 | 2467 (90) | 66 | - | 2668 | Elisa | 1373 | 50.22 | ***Low*** |
| Djoudi F. et al | 2023 | Retrospective | Algeria | 2010-2019 | 140168 | 140168 | 111461 (79.52) | - | - | - | 3rd Gen ELISA | 143 | 0.10 | ***Low*** |
| Singogo E. et al | 2023 | Retrospective | Malawi | 2015-2021 | 204920 | 204920 | 158508 (77.4) | - | - | - | ULTRA HBV EIA | 809 | 0.39 | ***Low*** |
| Gadji M. et al | 2024 | Prospective | Senegal | 2019-2021 | 5002 | 5002 | 3746 (75) | 2303 | - | 2699 | Chemiflex TM | 218 | 4.36 | ***Moderate*** |
| Jacobs G. et al | 2023 | Retrospective | South Africa | 2012-2016 | 515945 | 515397 | 228059 (44.3) | 515397 | - | - | NAT and InnoLIA | 3433 | 0.67 | ***Low*** |
| Koura M. et al | 2017 | Retrospective | Burkina Faso | 2009-2014 | 12969 | 12969 | 9797 (75.5) | - | - | - | Monolisa AgHBS | 535 | 4.13 | ***Moderate*** |
| Garba B. et al | 2023 | Retrospective | Nigeria | - | 400 | 400 | 400 (100) | - | - | - | LabACON | 46 | 11.50 | ***Low*** |

Enzyme-Linked Immunosorbent Assay (ELISA), *gen* generation, EIA based rapid immunochromatographic rapid kits, rapid diagnostic tests (RDTs), Hepatitis B Vírus (HBV), Hepatitis B surface antigen (HBsAg), HCV- Hepatitis C Virus InnoLIA (Innogenetics), Human Immunodeficiency Vírus (HIV), Real-time polymerase chain reaction (RT-PCR) assay; - Treponema pallidum Haemaglutination Assay (*TPHA*) test; Veneral Disease Research Laboratory (VDRL) test and**,** Plasmin Reagin Test (RPR), DiaSpot (cassette type), ACON (strips) and oneStep HIGHTOP (strips), rapid test kit (LabACON) for the qualitative detection of HBsAg.
