# Supplementary material for: Seroprevalence of Hepatitis B virus surface antigen among African blood donors: a systematic review and meta-analysis
Source: Front Public Health. 2024 Oct 21;12:1434816. doi: 10.3389/fpubh.2024.1434816 (PMC11532187; doi:10.3389/fpubh.2024.1434816)
Supplement: Supplementary file 3 [file Table_3.DOCX]

**Supplementary materials 3**

**Excluded references**

1. **Systematic reviews**

- Abdel-Gawad, M., et al., Gender differences in prevalence of hepatitis C virus infection in Egypt: a systematic review and meta-analysis. Sci Rep, 2023. 13(1): p. 2499.
- Agyeman, A.A., et al., Epidemiology of hepatitis C virus in Ghana: a systematic review and meta-analysis. BMC Infect Dis, 2016. 16: p. 391.
- Ajuwon, B.I., et al., Hepatitis B virus infection in Nigeria: a systematic review and meta-analysis of data published between 2010 and 2019. BMC Infect Dis, 2021. 21(1): p. 1120.
- Azevedo, T.C., et al., Hepatitis C in HIV-infected individuals: a systematic review and meta-analysis of estimated prevalence in Africa. J Int AIDS Soc, 2016. 19(1): p. 20711.
- Badawi, M.M., M.S. Atif, and Y.Y. Mustafa, Systematic review and meta-analysis of HIV, HBV and HCV infection prevalence in Sudan. Virol J, 2018. 15(1): p. 148.
- Belyhun, Y., et al., Hepatitis viruses in Ethiopia: a systematic review and meta- analysis. BMC Infect Dis, 2016. 16(1): p. 761.
- Chaabna, K., S.P. Kouyoumjian, and L.J. Abu-Raddad, Hepatitis C Virus Epidemiology in Djibouti, Somalia, Sudan, and Yemen: Systematic Review and Meta-Analysis. PLoSOne, 2016. 11(2): p. e0149966.
- De Buck, E., et al., Is having sex with other men a risk factor for transfusion transmissible infections in male blood donors in Western countries? A systematic review. PLoS One, 2015. 10(4): p. e0122523.
- Farahmand, M., et al., Molecular and serological markers of human parvovirus B19 infection in blood donors: A systematic review and meta-analysis. Asian J Transfus Sci, 2021. 15(2): p. 212-222.
- Fite, R.O., et al., Seroprevalence and factors associated with hepatitis B virus infection in blood donors in Ethiopia: a systematic review and meta-analysis. Arch Virol, 2020. 165(5): p. 1039-1048.
- Fox, J.M., et al., Seroprevalence of HTLV-1 and HTLV-2 amongst mothers and children in Malawi within the context of a systematic review and meta-analysis of HTLV seroprevalence in Africa. Trop Med Int Health, 2016. 21(3): p. 312-24.
- Geremew, D., et al., Seroprevalence of HIV among pregnant women in Ethiopia: a systematic review and meta-analysis. BMC Res Notes, 2018. 11(1): p. 908.
- Geremew, H. and D. Geremew, Sero-prevalence of syphilis and associated factors among pregnant women in Ethiopia: a systematic review and meta-analysis. Syst Rev, 2021. 10(1): p. 223.
- Im, Y.R., et al., Prevalence of occult hepatitis B virus infection in adults: a systematic review and meta-analysis. Lancet Gastroenterol Hepatol, 2022. 7(10): p. 932-942.
- Kebede, K.M., et al., The epidemiology of syphilis in Ethiopia: a protocol for systematic review and meta-analysis covering the last three decades. Syst Rev, 2019. 8(1): p. 210.
- Laermans, J., et al., Impact of disasters on blood donation rates and blood safety: A systematic review and meta-analysis. Vox Sang, 2022. 117(6): p. 769-779.
- Lake, E.A., et al., Seroprevalence of hepatitis c virus infection among blood donors in Ethiopia: a systematic review and meta-analysis. BMC Infect Dis, 2021. 21(1): p. 131.
- Makokha, G.N., et al., The burden of Hepatitis B virus infection in Kenya: A systematic review and meta-analysis. Front Public Health, 2023. 11: p. 986020.
- McNaughton, A.L., et al., Hepatitis B virus seroepidemiology data for Africa: Modelling intervention strategies based on a systematic review and meta-analysis. PLoS Med, 2020. 17(4): p. e1003068.
- Mulugeta, H., et al., Seroprevalence and trend of human immunodeficiency virus among blood donors in Ethiopia: a systematic review and meta-analysis. BMC Infect Dis, 2019. 19(1): p. 383.
- Musa, B.M., et al., Prevalence of hepatitis B virus infection in Nigeria, 2000-2013: a systematic review and meta-analysis. Niger J Clin Pract, 2015. 18(2): p. 163-72.
- Muzembo, B.A., et al., Systematic review and meta-analysis of hepatitis C virus infection in the Democratic Republic of Congo. Public Health, 2016. 139: p. 13-21.
- Ngoma, A.M., et al., Seroprevalence of human T-lymphotropic virus (HTLV) in blood donors in sub-Saharan Africa: a systematic review and meta-analysis. Vox Sang, 2019. 114(5): p. 413-425.
- Noori, M., et al., ABO blood groups and risk of human immunodeficiency virus infection: A systematic review and meta-analysis. Rev Med Virol, 2022. 32(3): p. e2298.
- Ofori-Asenso, R. and A.A. Agyeman, Hepatitis B in Ghana: a systematic review & meta-analysis of prevalence studies (1995-2015). BMC Infect Dis, 2016. 16: p. 130.
- Riou, J., et al., Hepatitis C virus seroprevalence in adults in Africa: a systematic review and meta-analysis. J Viral Hepat, 2016. 23(4): p. 244-55.
- Tafesse, T.B., et al., Seroprevalence and diagnosis of HIV, HBV, HCV and syphilis infections among blood donors. Hum Antibodies, 2017. 25(1-2): p. 39-55.
- Yazie, T.D. and M.G. Tebeje, An updated systematic review and meta-analysis of the prevalence of hepatitis B virus in Ethiopia. BMC Infect Dis, 2019. 19(1): p. 917. 561
- Kwange, S.O., et al., Hepatitis B virus subgenotype A1, occurrence of subgenotype D4, and S gene mutations among voluntary blood donors in Kenya. Virus Genes, 2013. 47(3): p. 448-55
- Wu, S., Wang, J., Guo, Q., Lan, H., Sun, Y., Ren, M., ... & Li, G. (2023). Prevalence of human immunodeficiency virus, syphilis, and hepatitis B and C virus infections in pregnant women: a systematic review and meta-analysis. *Clinical Microbiology and Infection*, *29*(8), 1000-1007.
- Geremew, D., et al., Seroprevalence of HIV among pregnant women in Ethiopia: a systematic review and meta-analysis. BMC Res Notes, 2018. 11(1): p. 908.
- Riou, J., et al., Hepatitis C virus seroprevalence in adults in Africa: a systematic review and meta-analysis. J Viral Hepat, 2016. 23(4): p. 244-55.
- Towns, J. M., Tieosapjaroen, W., Mello, M. B., Baggaley, R. C., Johnson, C. C., Jamil, M. S., ... & Ong, J. J. (2023). The role of syphilis self-testing as an additional syphilis testing approach in key populations: a systematic review and meta-analysis. *The Lancet Public Health*.
- Schroyens, N., Borra, V., Compernolle, V., Vandekerckhove, P., & De Buck, E. (2023). Men who have sex with men and risk for transfusion‐transmissible infections in blood donors in Western countries: A systematic review update. *Vox Sanguinis*, *118*(9), 709-720.
- Puerto‐Meredith, S., Singogo, E., Chagomerana, M., Nthani, T., Likaka, A., Gondwe, A., ... & Hosseinipour, M. C. (2023). Systematic review of prevalence and risk factors of transfusion transmissible infections among blood donors, and blood safety improvements in Southern Africa. *Transfusion Medicine*, *33*(5), 355-371.
- Makokha, G. N., Zhang, P., Hayes, C. N., Songok, E., & Chayama, K. (2023). The burden of Hepatitis B virus infection in Kenya: A systematic review and meta-analysis. *Frontiers in public health*, *11*, 986020.
- Kafeero, H. M., Ndagire, D., Ocama, P., Kato, C. D., Wampande, E., Walusansa, A., ... & Sendagire, H. (2023). Mapping hepatitis B virus genotypes on the African continent from 1997 to 2021: A systematic review with meta-analysis. *Scientific Reports*, *13*(1), 5723.
- Azzam, A., Khaled, H., El-Kayal, E. S., Gad, F. A., & Omar, S. (2023). Prevalence of occult hepatitis B virus infection in Egypt: a systematic review with meta-analysis. *Journal of the Egyptian Public Health Association*, *98*(1), 13.

**B: Did not study the prevalence of HBV among blood donors**

- Martinson, F.E., et al., Risk factors for horizontal transmission of hepatitis B virus in a rural district in Ghana. Am J Epidemiol, 1998. 147(5): p. 478-87.
- deMedina, M.D., et al., Prevalence of anti-HIV and anti-delta among deferred HBsAg- positive volunteer blood donors. Ann Intern Med, 1987. 107(2): p. 256-7.
- Mabunda, N., et al., Molecular and serological characterization of occult hepatitis B among blood donors in Maputo, Mozambique. Mem Inst Oswaldo Cruz, 2020. 115: p. e200006.
- Fopa, D., et al., Occult hepatitis B infection among blood donors from Yaoundé, Cameroon. Blood Transfus, 2019. 17(6): p. 403-408.
- Nébié, K., et al., Poor procedures and quality control among nonaffiliated blood centers in Burkina Faso: an argument for expanding the reach of the national blood transfusion center. Transfusion, 2011. 51(7 Pt 2): p. 1613-8.
- El-Nabi, S.H., et al., Anti-HBc and hepatitis B virus DNA among HBsAg-negative blood donors from the main central blood bank units in Eastern Libya. Transfus Med, 2020. 30(5): p. 401-405.
- Sanou, A.M., et al., Prevalence of Hepatitis B virus and Hepatitis D virus Coinfection in Western Burkina Faso and molecular characterization of the detected virus strains. Int J Infect Dis, 2018. 70: p. 15-19.
- Funeh, C.N., et al., Seroprevalence of hepatitis B virus among people screened at a primary care hospital in Bamenda: a cross-sectional study. Pan Afr Med J, 2022. 41: p. 237.
- Oluyinka, O.O., et al., Occult Hepatitis B Virus Infection in Nigerian Blood Donors and Hepatitis B Virus Transmission Risks. PLoS One, 2015. 10(7): p. e0131912.
- Zahn, A., et al., Molecular characterization of occult hepatitis B virus in genotype E- infected subjects. J Gen Virol, 2008. 89(Pt 2): p. 409-418.
- Fujiwara, K., et al., Distribution of HBV genotypes among HBV carriers in Benin:phylogenetic analysis and virological characteristics of HBV genotype E. World J Gastroenterol, 2005. 11(41): p. 6410-5.
- Darwish, N.M., et al., Hepatitis C virus infection in blood donors in Egypt. J Egypt Public Health Assoc, 1992. 67(3-4): p. 223-36.
- Dray, X., et al., [Prevalences of HIV, hepatitis B and hepatitis C in blood donors in the Republic of Djibouti]. Med Trop (Mars), 2005. 65(1): p. 39-42.
- Sumbu, B.M.M., et al., Epidemiological Surveillance of Blood Donors in Kinshasa, 2014 - 2018. Clin Lab, 2022. 68(10).
- Zeba, M.T., et al., Prevalence of HBV and HCV markers among patients attending the Saint Camille Medical Centre in Ouagadougou. Pak J Biol Sci, 2012. 15(10): p. 484- 9.
- Nnakenyi, I.D., C. Uchechukwu, and U. Nto-Ezimah, Prevalence of hepatitis B and C virus co-infection in HIV positive patients attending a health institution in southeast Nigeria. Afr Health Sci, 2020. 20(2): p. 579-586.
- Katamba, C., T. Chungu, and C. Lusale, HIV, syphilis and hepatitis B coinfections in Mkushi, Zambia: a cross-sectional study. F1000Res, 2019. 8: p. 562.
- Allain, J.P., et al., Hepatitis B Virus Chronic Infection in Blood Donors from Asian and African High or Medium Prevalence Areas: Comparison According to Sex. Viruses, 2022. 14(4).
- Toyé, R.M., et al., Prevalence and molecular characterization of hepatitis B virus infection in HIV-infected children in Senegal. Clin Res Hepatol Gastroenterol, 2021. 45(2): p. 101502.
- Mogtomo, M.L., et al., [Screening of infectious microorganisms in blood banks in Douala (1995-2004)]. Sante, 2009. 19(1): p. 3-8.
- Chasela, C.S., et al., Prevalence of hepatitis C virus infection among human immunodeficiency virus-1-infected pregnant women in Malawi: the BAN study. J Clin Virol, 2012. 54(4): p. 318-20.
- Jobarteh, M., et al., Seroprevalence of hepatitis B and C virus in HIV-1 and HIV-2 infected Gambians. Virol J, 2010. 7: p. 230.
- Jayaraman, S., et al., The risk of transfusion-transmitted infections in sub-Saharan Africa. Transfusion, 2010. 50(2): p. 433-42.
- Sem criterios
- Jahun, M. M., Rogo, L. D., Ibrahim, A., Sa'id, H., Isah, S. Y., Kumurya, A. S., & Amadu, M. (2018). Human T Cell lymphotropic virus types 1 and 2 among blood donors in Aminu Kano Teaching Hospital. *World J. Biomed. Res.(Online)*, 1-5.

**C: Pregnant women**

- Vueba, A.N., et al., Prevalence of HIV and hepatitis B virus among pregnant women in Luanda (Angola): geospatial distribution and its association with socio-demographic and clinical-obstetric determinants. Virol J, 2021. 18(1): p. 239.
- Dionne-Odom, J., et al., Hepatitis B, HIV, and Syphilis Seroprevalence in Pregnant Women and Blood Donors in Cameroon. Infect Dis Obstet Gynecol, 2016. 2016: p. 4359401.
- Bejide, I. O., Kayode, T. A., Ebagua, A. E., Obayendo, O. D., Mere, D. O., Newman, A. B., ... & Folarin, O. A. (2024). Seroprevalence of Hepatitis B virus and human immunodeficiency virus co-infection in pregnant women from Osun State, Nigeria. *The Journal of Infection in Developing Countries*, *18*(01), 145-151.
- Anabire, N. G., Quaye, O., & Helegbe, G. K. (2023). Circulation of multiple hepatitis B virus genotypes in individual pregnant women seeking antenatal care in northern Ghana. *Virology Journal*, *20*(1), 149.
- Abuku, V. G., Allotey, E. A., & Akonde, M. (2023). Clinical and laboratory presentation of first-time antenatal care visits of pregnant women in Ghana, a hospital-based study. *Plos one*, *18*(1), e0280031.
- Aguiar, D. M. D., de Andrade, A. M., Ramalho, A. A., Martins, F. A., Koifman, R. J., Opitz, S. P., & da Silva, I. F. (2023). Effect of prenatal care quality on the risk of low birth weight, preterm birth and vertical transmission of HIV, syphilis, and hepatitis. *PLOS Global Public Health*, *3*(3), e0001716.
- Dude, A. M., Drexler, K., Yee, L. M., & Badreldin, N. (2023). Adherence to Sexually Transmitted Infection Screening in Pregnancy. *Journal of Women's Health*, *32*(6), 652-656.
- Kuugbee, Eugene D., Gloria Maaldu, Aseta Adamu, Nafisa Salia, Williams Walana, Sylvanus Kampo, Ezekiel K. Vicar, and Juventus B. Ziem. "Seroprevalence and Risk Factors of Sexually Transmitted Blood‐Borne Infections among Pregnant Women Attending Antenatal Care in Jirapa, Upper West Region of Ghana." *Canadian Journal of Infectious Diseases and Medical Microbiology* 2023, no. 1 (2023): 3157202.
- Shepard, D. S., Halasa-Rappel, Y. A., Rowlands, K. R., Kulchyckyj, M., Basaza, R. K., Otieno, E. D., ... & Musange, S. F. (2023). Economic analysis of a new four-panel rapid screening test in antenatal care in Kenya, Rwanda, and Uganda. *BMC health services research*, *23*(1), 815.

**D: Child**

- Suesstrunk, J. and F.B. Djongali, Hepatitis B virus prevalence in rural areas in south-west Chad. Trop Doct, 2017. 47(4): p. 374-377.
- Martinson, F.E., et al., Risk factors for horizontal transmission of hepatitis B virus in a rural district in Ghana. Am J Epidemiol, 1998. 147(5): p. 478-87.
- Allain, J.P., et al., Hepatitis B Virus Chronic Infection in Blood Donors from Asian and African High or Medium Prevalence Areas: Comparison According to Sex. Viruses, 2022. 14(4).
- Dongdem, J.T., et al., Prevalence of hepatitis B virus infection among blood donors at the Tamale Teaching Hospital, Ghana (2009). BMC Res Notes, 2012. 5: p. 115.
- Seroprevalence of hepatitis B virus among people screened at a primary care hospital in Bamenda: a cross-sectional study.Cyprine Neba Funeh, Peter Vanes Ebasone, Eric Mbah Chunga, Fonyuy Nkwawir, Rogers Ajeh, Blaise Barche, Ignatius Fonyong Tebid. Cyprine Neba Funeh et al. PAMJ - 41(237). 22 Mar 2022. Available online at: https://www.panafrican-med-journal.com//content/article/41/237/full
- Prevalence of viral hepatitis B markers among blood donors in the Republic of Guinea Sanaba Boumbaly, Thierno Amadu Labe Balde, AlexandrV.Semenov,Yulia V. Ostankova, Elena N. Serikova, Ekaterina V. Naidenova, Diana E. Valutite, Alexandr N. Shchemelev, Elena B. Zueva, Elena V. Esaulenko, Areg A. Totolian.DOI: <https://doi.org/10.36233/0507-4088-92>.
- Funeh, C.N., et al., Seroprevalence of hepatitis B virus among people screened at a primary care hospital in Bamenda: a cross-sectional study. Pan Afr Med J, 2022. 41: p. 237.
- Toyé, R.M., et al., Prevalence and molecular characterization of hepatitis B virus infection in HIV-infected children in Senegal. Clin Res Hepatol Gastroenterol, 2021. 45(2): p. 101502.
- Mogtomo, M.L., et al., [Screening of infectious microorganisms in blood banks in Douala (1995-2004)]. Sante, 2009. 19(1): p. 3-8.
- Hepatitis B virus prevalence in rural áreas in south-west Chad Julian Suesstrunk1 and Fe´de´ric Berniba Djongali. Tropical Doctor 2017, Vol. 47(4) 374–377sagepub.co.uk/journalsPermissions.nav DOI: 10.1177/0049475517699718. journals.sagepub.com/home/todo.
- Risk Factors for Horizontal Transmission of Hepatitis B Virus in a RuralDistrict in Ghana, Francis E. A. Martinson, Kristen A. Weigle, Rachel A. Royce, David J. Weber, Chirayath M. Suchindran, and Stanley M. Lemon American Journal of Epidemiology Copyright C 1998 by The Johns Hopkins University School of Hygiene and Public HealthVol. 147, No. 5 .Printed in U.S.A
- 10-Prevalence of hepatitis B virus infection among blood donors at the Tamale Teaching Hospital, Ghana (2009) , Julius Tieroyaare Dongdem1*, Sylvanus Kampo, Ireneous N Soyiri, Patrick Nsobila Asebga, Juventus B Ziem, and Kenneth Sagoe Dongdem et al. BMC Research Notes 2012, 5:115 <http://www.biomedcentral.com/1756-0500/5/115> doi:10.1186/1756-0500-5-115.

**E: Without relevant data**

- Boumbaly, S., et al., [Prevalence of viral hepatitis B markers among blood donors in the Republic of Guinea]. Vopr Virusol, 2022. 67(1): p. 59-68.
- Darwish, N.M., et al., Hepatitis C virus infection in blood donors in Egypt. J Egypt Public Health Assoc, 1992. 67(3-4): p. 223-36.
- Dray, X., et al., [Prevalences of HIV, hepatitis B and hepatitis C in blood donors in the Republic of Djibouti]. Med Trop (Mars), 2005. 65(1): p. 39-42.
- Sumbu, B.M.M., et al., Epidemiological Surveillance of Blood Donors in Kinshasa, 2014 - 2018. Clin Lab, 2022. 68(10).
- Zeba, M.T., et al., Prevalence of HBV and HCV markers among patients attending the Saint Camille Medical Centre in Ouagadougou. Pak J Biol Sci, 2012. 15(10): p. 484-9.
- Serological findings amongst first-time blood donors in Yaoundé, Cameroon: is safe donation a reality or a myth? D N Mbanya 1, D Takam, P M Ndumbe. Transfus Med 2003 Oct;13(5):267-73.doi: 10.1046/j.1365-3148.2003.00453.x.
- Évaluation du risque infectieux résiduel chez les donneurs de sang au Centre national de transfusion sanguine de Conakry.
- A Loua 1, E M L Sow, F B Magassouba, M Camara, M A Baldé. PMID: 15120107 doi: 10.1016/j.tracli.2004.01.004
- Risque résiduel de transmission du VIH, VHB et VHC par transfusion sanguine entre 2002 et 2004 au Centre national de transfusion sanguine d’Abidjan. A O Touré-Fall 1, T N D Dièye, A Sall, M Diop, M Seck, S Diop, D Thiam, L Diakhaté. Transfus Clin Biol. 2009 Nov-Dec;16(5-6):439-43. doi: 10.1016/j.tracli.2009.09.005.
- Prevalence of Hepatitis B virus and Hepatitis D virus Coinfection in Western Burkina Faso and molecular characterization of thedetected virus strainsArmel M. Sanoua,* Kenza Benkiraneb, Bachirou Tintoc, Assana Cisséa, Tani Sagnaa, Abdoul Kader Ilboudoa, Claire Dordingb, Zekiba Tarnagdaa, Claude P. Mullerb,Judith M. Hübschenbhttps://doi.org/10.1016/j.ijid.2018.02.004.
- Hepatitis B virus subgenotype A1, occurrence of subgenotype D4,and S gene mutations among voluntary blood donors in KenyaSimeon Owuor Kwange • Nancy L. M. Budambula •Michael Kibet Kiptoo • Fredrick Okoth • Missiani Ochwoto •Margaret Oduor • James Hungo Kimotho.Virus Genes (2013) 47:448–455 DOI 10.1007/s11262-013-0976-1.
- Prevalence Du Vih Et L’antigene Hbs Chez Les Donneurs Du Sang. Risque Residuel De Contamination Chez Les Receveurs De Sang A Kinshasa-Est.Republique Democratique Du Congo C.Mbendi Nlombi, B.Longo-Mbenza, S.Mbendi Nsukini, J.J.Muyembe Tamfum, H.Situakibanza Nanituma, D.Vangu Ngoma Med.Trop.2001;61:139-142
- Poor procedures and quality control among non-affiliated blood centers in Burkina Faso: an argument for expanding the reach of the national blood transfusion center Koumpingnin Nébié, Siaka Ouattara, Mahamoudou Sanou, Youssouphe Kientega, Honorine Dahourou, Lassina Ky, Kisito Kienou1, Samba Diallo, Françoise Bigirimana, Catherine Fretz, Edward L. Murphy, and Jean-Jacques Lefrère. Transfusion.2011July; 51(7PT2): 1613–1618. doi:10.1111/j.1537-2995.2011.03222.x.
- Risque résiduel de la transmission par transfusion de l´infection du virus de l´hépatite B dû aux donneurs porteurs de l´infection du virus de l´hépatite B occulte à Yaoundé, CamerounMichel Kengne, Yolande Francine Onomo Medja, Tedom, Julius Mbekem Nwobegahay Pan African Medical Journal. 2021;39(175). 10.11604/pamj.2021.39.175.22365. Available online at: <https://www.panafrican-med-journal.com//content/article/39/175/full>.
- A multiplexed protein microarray for the simultaneous serodiagnosis of human immunodeficiency virus/hepatitis C virus infection and typing of whole blood. Stewart T.G. Burgess, Fiona Kenyon, Nichola O’Looney, Alan J. Ross, Marisa Chong Kwan, John S. Beattie, Juraj Petrik, Peter Ghazal, Colin J. Campbell. doi:10.1016/j.ab.2008.07.017.
- Prevalence of serum antibodies against bloodborne and sexually transmitted agents in selected groups in Somalia, Y. A. NUR, J. GROEN, A. M. ELM I, A. OTT, A. D. M. E. OSTERHAUS.Epidemiol. Infect. (2000), 124, 137–141.

**F: Article not found full text**

- Infectious markers among blood donors in Democratic Republic of Congo (DRC)]. Rev Med Brux May-Jun 2007;28(3):145-9.A Batina 1, S Kabemba, R Malengela.
- Seroprevalence and epidemiological profile of blood donors at the Lubumbashi university clinics, Democratic Republic of Congo | Profil épidémiologique et séroprévalence des donneurs de sang aux cliniques universitaires de Lubumbashi, République Démocratique du Congo.
- Seroprevalence of hepatitis B and C and of human immunodeficiency virus among blood donors in south-west Nigeria, V O Mabayoje 1, D P Oparinde, E O Akanni, S S Taiwo, M A Muhibi, T O Adebayo Br J Biomed Sci No abstract available. 2007;64(4):177-9. doi: 10.1080/09674845.2007.11978104.
- [Prevalence of serum markers of hepatitis B and C virus in blood donors of Nouakchott, Mauritania] B B Lo 1, M Meymouna, M A Boulahi, M Tew, A Sow, A Ba, M B Sow. Bull Soc Pathol Exot.1999 May;92(2):83-4.
- Seroprevalence of hepatitis B and C and of human immunodeficiency virus among blood donors in south-west Nigeria.V O Mabayoje 1, D P Oparinde, E O Akanni, S S Taiwo, M A Muhibi, T O Adebayo doi:10.1080/09674845.2007.11978104

**G: Included patients already positive for hepatitis B**

- Zeba, M.T., et al., Prevalence of HBV and HCV markers among patients attending the Saint Camille Medical Centre in Ouagadougou. Pak J Biol Sci, 2012. 15(10): p. 484-9. DOI:10.3923/pjbs.2012.484.489
- Nnakenyi, I.D., C. Uchechukwu, and U. Nto-Ezimah, Prevalence of hepatitis B and C virus co-infection in HIV positive patients attending a health institution in southeast Nigeria. Afr Health Sci, 2020. 20(2): p. 579-586.
- Katamba, C., T. Chungu, and C. Lusale, HIV, syphilis and hepatitis B coinfections in Mkushi, Zambia: a cross-sectional study. F1000Res, 2019. 8: p. 562.
- Allain, J.P., et al., Hepatitis B Virus Chronic Infection in Blood Donors from Asian and African High or Medium Prevalence Areas: Comparison According to Sex. Viruses, 2022. 14(4).
- Toyé, R.M., et al., Prevalence and molecular characterization of hepatitis B virus infection in HIV-infected children in Senegal. Clin Res Hepatol Gastroenterol, 2021. 45(2): p. 101502.
- Sanou, A.M., et al., Prevalence of Hepatitis B virus and Hepatitis D virus Coinfection in Western Burkina Faso and molecular characterization of the detected virus strains. Int J Infect Dis, 2018. 70: p. 15-19.
- Nnakenyi, I.D., C. Uchechukwu, and U. Nto-Ezimah, Prevalence of hepatitis B and C virus co-infection in HIV positive patients attending a health institution in southeast Nigeria. Afr Health Sci, 2020. 20(2): p. 579-586
- HIV, syphilis and hepatitis B coinfections in Mkushi, Zambia: a cross-sectional study.Cibangu Katamba 1, Theresa Chungu, Chisali Lusale, https://doi.org/10.12688/f1000research.17983.1
- HIV, syphilis and hepatitis B coinfections in Mkushi, Zambia: a cross-sectional study.Cibangu Katamba 1, Theresa Chungu, Chisali Lusale Latest published: 27 Jul 2020, 8:562 <https://doi.org/10.12688/f1000research.17983.2>
- Prevalence of hepatitis B and C virus co-infection in HIV positive patients attending a health institution in southeast Nigeria, Ifeyinwa Dorothy Nnakenyi, Chisom Uchechukwu, Uloaku Nto-ezimah, Afri Health Sci.2020; 20(2): 579-586. <https://doi.org/10.4314/ahs.v20i2.5> .
- Occult hepatitis B infection among blood donors from Yaoundé, Cameroon, Diderot Fopa, Daniel Candotti, Claude T. Tagny, Camille Doux, Dora Mbanya, Edward L. Murphy, Hany I. Kenawy, Farha El Chenawi, Syria Laperche. Blood Transfus 2019; 17: 403-8 DOI 10.2450/2019.0182-19.
- Occult Hepatitis B Virus Infection in Nigerian Blood Donors and Hepatitis B Virus Transmission Risks Opaleye O. Oluyinka, Hoang Van Tong, Sy Bui Tien, Ademola H. Fagbami, Olusegun Adekanle, Olusola Ojurongbe, C.-Thomas Bock, Peter G. Kremsner, Thirumalaisamy P. Velavan. PLoS ONE 10(7): e0131912doi:10.1371/journal.pone.0131912
